# Risk factors for infections after urological procedures among patients with negative urine culture screening

**DOI:** 10.1017/ash.2025.10271

**Published:** 2026-01-20

**Authors:** Nutnicha Tantiwattanapaibul, Anucha Apisarnthanarak, Patranuch Noppakulsatit, Chatchawet Liwrotsap, Teerayut Tangpaitoon, Valeerat Swatesutipun, Dollacha Vanichakarn, Natthapitch Tangkaew, Kittiya Jantarathaneewat, Nuntra Suwantarat

**Affiliations:** 1 Division of Infectious Disease, Department of Medicine, Faculty of Medicine, Thammasat University, Pathum Thani, Thailand; 2 Division of Infectious Disease, Department of Medicine, Thammasat University Hospital, Pathum Thani, Thailand; 3 Research Group in Infectious Disease Epidemiology and Prevention, Faculty of Medicine, Thammasat University, Pathum Thani, Thailand; 4 Division of Urology, Department of Surgery, Faculty of Medicine, Thammasat University, Pathum Thani, Thailand; 5 Center of Excellence in Pharmacy Practice and Management Research, Faculty of Pharmacy, Thammasat University, Pathum Thani, Thailand; 6 Chulabhorn International College of Medicine, Thammasat University, Pathum Thani, Thailand

## Abstract

In this retrospective cohort, chronic kidney disease stage III-V (*P* = .005), history of previous urinary tract infection within 3 months (*P* < .05), Revised Cardiac Risk Index for preoperative risk ≥2 (*P* = .002), and percutaneous nephrolithotomy procedure (*P* < .05) were associated with risks of infections after urological procedures among patients with negative urine culture screening.

## Introduction

The rise in urological diseases had resulted in an increased demand for urological diagnostic procedures and treatment interventions. Postoperative infections had become significant complications, with reported rates ranging from 1.7% to 18.8% for the upper urinary tract related procedures including percutaneous nephrolithotomy (PCNL), ureteroscopy (URS), and retrograde intrarenal surgery (RIRS), and 2.6% to 13.5% for the lower urinary tract related procedures including transrectal ultrasound (TRUS) and biopsy of the prostate, transurethral resection of the prostate (TURP), holmium laser enucleation of the prostate (HoLEP), and transurethral resection of bladder tumor (TURBT).^1–4^ Among the infections after prostatic procedures, the most prevalent pathogens were *Escherichia coli* (15.97%), *Pseudomonas aeruginosa* (10.76%), and *Enterococcus faecalis* (9.72%), where the most prevalent pathogens for PCNL were *Escherichia coli* (43.9%), *Enterococcus faecalis* (9.2%), and *Klebsiella pneumoniae* (5.6%).^1–6^ In addition, the increase prevalence of extended spectrum beta-lactamase (ESBL) or extended spectrum cephalosporinase (ESC) pathogens infections had been in arising in the Southeast Asia, where the prevalence of ESBL/ESC infections after urological procedures were reported of 59.5%.^7,8^ Risk factors for ESBL/ ESC infections have been identified such as a history of antibiotic use in the past 3 months, recent hospital admissions, prior urinary tract infections (UTIs), and positive urine culture screening without clinical signs of urinary tract infections before the procedures (asymptomatic bacteriuria).^2–9^ The consequences of postoperative infections resulted in prolonged hospital stays, multidrug-resistant (MDR) bacterial infections, and increased costs of treatment.

Given the ongoing challenges of postoperative infections, this study aimed to identify specific risk factors contributing to infections after elective urological procedures in patients with negative urine culture screening. Furthermore, the study results would be determined the rate of the infection and provided valuable insights to improve preoperative management and patient outcomes especially in the endemic area of MDR bacterial infections.

## Methods

We conducted a retrospective cohort study, including patients aged ≥18 years who underwent elective urological procedures (TRUS and biopsy of the prostate, TURP, HoLEP, PCNL, URS, RIRS, and TURBT) at Thammasat University Hospital (TUH), a 750-beds tertiary care hospital, Thailand. The study patients were identified from ICD-9 of urological procedures between January 1, 2021 and September 30, 2023. The electronic medical records of all patients were reviewed for demographic characteristics, underlying medical diseases, history of recent hospital admissions, antibiotic use and prior UTIs within 3 months, prior urological procedures within the past year, retention of Foley catheter prior to the procedure, American Society of Anesthesiologists (ASA) physical status classification system, Metabolic equivalents (METS) score, Revised Cardiac Risk Index (RCRI) for preoperative risk, type of antibiotic prophylaxis, type and duration of the urological procedure, volume of the blood loss, postintervention infections, microbiological culture results, length of hospital stay and follow-up visit. As the urological procedure protocol, all patients had urological evaluation and urine culture screening prior to the procedures. For the patients with negative urine culture screening, oral levofloxacin would be prescribed as antibiotic prophylaxis for TRUS and biopsy of the prostate and intravenous cefoxitin would be prescribed for other urological procedures. Posturological procedure complications were included UTI and sepsis/bacteremia. UTI was defined as patient experiencing dysuria with or without fever or suprapubic pain, with a urine culture positive for ≥10^5^ colony-forming units/ml and no more than two species of uropathogenic microorganisms. Sepsis was diagnosed if they met two or more Systemic Inflammatory Response Syndrome criteria. Bacteremia was diagnosed if blood culture revealed microorganisms compatible with uropathogenic microorganisms.

The descriptive data were presented in number (percentage) and mean with standard deviation or median with interquartile range (IQR) depended on data distribution. Percentages were compared using *χ* ^2^ test or Fisher exact test, while the continuous data using Mann–Whitney *U* test. Multivariable logistic regression analysis was performed to determine risk factor independent and outcome variables. Statistical analyses were performed using SPSS version 22. This study was approved by the Ethics Committee of Thammasat University (MTU-EC-IM-0-280/66).

## Results

There were 441 patients included in this study. In the study period, 55 patients (12.5%) were infected following urological procedures, while 386 patients remained uninfected. The study population had a median age of 68 years (IQR 61–74) with 356 males (80.7%). The most common comorbidities were cardiovascular disease (54%), benign prostatic hyperplasia (49.7%), and dyslipidemia (40.6%). There were 173 patients (39.2%) with a history of renal stones and 119 patients (27%) with a history previous intervention within 3 months. For the other risks of MDR bacterial infections, there were patients with previous antibiotics used in the last 3 months (8.2%), previous UTI within the last 3 months (7.9%), previous admission in the last 3 months (1.4%) and a history of foley catheter retention before procedure (1.4%). The median time from operation to the infection onset was 6 days (IQR 5–7). The median ASA physical status classification was class 2 (70.7%). The median RCRI for preoperative risk was zero (84.1%). The mean operation time was 103 minutes (IQR 70–130). The most common interventions were TRUS and biopsy of prostate (26.5%), TURBT (15.4%), and URS (13.8%) (Table 1).

**Table 1.** Patients characteristics

Characteristics	Total (*n* = 441)	Infected patients (*n* = 55)	Non-infected patients (*n* = 386)	*P*-value
Age (year, median, IQR)	68 (61–74)	70 (64–77)	68 (60–73)	.047
Male sex	356 (80.7)	39 (70.9)	317 (82.1)	.049
Medical comorbidity Benign prostatic hyperplasia Cardiovascular disease Diabetes mellitus Malignancy^a^ Chronic kidney disease stage III–V	219(49.7) 238 (54) 116 (26.3) 32 (7.3) 26 (5.9)	23 (41.8) 33 (60) 21 (38.2) 5 (9.1) 12 (21.8)	196 (50.8) 205 (53.1) 95 (24.6) 27 (7) 14 (3.6)	214 .337 .032 .575 <.05
History Renal stones Urological interventions^b^ Antibiotic use in past 3 months Prior UTI in past 3 months Admission in past 3 months Retention of Foley catheter	173 (39.2) 119 (27) 36 (8.2) 35 (7.9) 6 (1.4) 6 (1.4)	31 (56.4) 17 (30.9) 12 (21.8) 12 (21.8) 3 (5.5) 3 (5.5)	142 (36.8) 102 (26.4) 24 (6.2) 23 (6) 3 (0.8) 3 (0.8)	.005 .483 <.05 <.05 .028 .028
American Society of Anesthesiologists physical status classification system of 2	312(70.7)	39 (70.9)	273 (70.7)	.978
Metabolic equivalents score >4	433 (98.2)	50 (90.9)	383 (99.2)	.001
Revised Cardiac Risk Index (RCRI) for operative risk ≥2	11 (2.5)	7 (12.7)	4 (1)	<.05
Blood loss ≥50 ml	63 (14.3)	18 (32.7)	45 (11.7)	<.05
Operative duration (min, median, IQR)	103 (70–130)	133 (120–155)	100 (70–120)	<.05
Intervention Transrectal ultrasound and biopsy of prostate Transurethral resection of the prostate Holmium laser enucleation of the prostate Transurethral resection of bladder tumor Ureteroscope Retrograde intrarenal surgery Percutaneous nephrolithotomy	117 (26.5) 47 (10.7) 36 (8.2) 68 (15.4) 61(13.8) 60 (13.6) 52 (11.8)	6 (10.9) 4 (7.2) 9 (16.4) 5 (9.1) 5 (9.1) 9 (16.4) 17 (30.9)	111 (28.8) 43 (11.1) 27 (7) 63 (16.3) 63 (16.3) 56 (14.5) 35 (9.1)	.005 .488 .018 .165 .276 .524 <.05
Complication after urological procedure Urinary tract infection Sepsis/bacteremia	55 (12.47) 9 (2.04)	55 (100) 9 (16.36)	0 (0) 0 (0)	<.0001 <.0001
Length of stay (day, median, IQR)	3 (3–4)	12 (10–15)	3 (3–4)	<.05

Note. IQR, interquartile range; UTI, urinary tract infection.

a Colorectal cancer; breast cancer; cholangiocarcinoma.

b Prior urological interventions within 3 months.

The antibiotic prophylaxis included levofloxacin (26.5%) and cefoxitin (73.5%). UTI occurred in 55 patients (12.5%) with 9 patients (2%) developing urosepsis. The most common microorganisms were *E. coli* (49%), *P. aeruginosa* (16.4%) and *K. pneumoniae* (12.7%). The pattern of MDR pathogens included ESC isolates (43.6%). The median hospital stay was 12 days (IQR 10–15) for patients with infection and 3 days (IQR 3–4) for non-infection. No fatalities were reported.

Multivariable logistic regression analysis revealed several significant risk factors for posturological procedure infections. Theses included RCRI for preoperative risk score ≥2 (OR = 10.46; 95% CI 2.36–46.28, *P* = .002), history of previous UTI within the last 3 months (OR = 4.95; 95% CI 2.05–11.97, *P* < .05), and presence of underlying chronic kidney disease (CKD) stage III–V (OR = 4.42; 95% CI 1.55–12.60, *P* = .005). The interventions associated with a higher risk of infections after urological procedure were PCNL (OR = 5.39; 95% CI 2.20–13.23, *P* < .05) (Table 2). Through multivariable logistic regression analysis of risk factor of ESC pathogens detection, there was no associations with any risk factors (Supplement 1).

**Table 2.** Multivariate analysis of risk factors for infection after urological procedures among patients with negative urine culture screening

	Multivariate analysis	
Variable	OR	*P*-value
Chronic kidney disease stage III–V	4.42 (1.55–12.60)	.005
History of prior UTI in past 3 months	4.95 (2.05–11.97)	<.05
Revised Cardiac Risk Index for operative risk ≥2	10.46 (2.36–46.28)	.002
Percutaneous nephrolithotomy	5.39 (2.20–13.23)	<.05

Note. UTI, urinary tract infection.

## Discussion

Our study had the novel finding and implications. First, we identified CKD as the important risk factor for infection following urological procedures. To our knowledge this risk factor had never been identified in the prior literature. Second, we also identified RCRI score as an important severity of illness to predict the risk for the infection. Third, our study highlighted the high prevalence of MDR bacterial infection after the procedures.

In previous studies, the risk factors associated with infection following urological procedures included a history of antibiotic usage, recent hospitalization, and urinary tract infection within the past 3 months.^3–9^ One study had indicated that acute kidney injury (AKI) following PCNL procedures was correlated with infection after the procedure. Interestingly, individual who develop AKI were increased likelihood of postoperative sepsis and extended stay in the intensive care unit.^10^ In operations with prolonged duration and comorbidities, there was an increased risk of postoperative UTI, especially among PCNL patients with a history of past UTI and Foley catheter use.^7–10^

In our study, the RCRI for preoperative risk ≥2 was an important risk factor of infection after urological procedure. The RCRI score incorporated factor such as the type of surgical procedure with high risk profile, history of ischemic heart disease, congestive heart failure, cerebrovascular disease, preoperative treatment with insulin, and preoperative creatinine >2 mg/dL/176.8 μmol/L. These factors predicted the perioperative risk of cardiac events and identified individuals with multiple underlying diseases in vulnerable groups, which could lead to a high risk of infection following procedures.

Similar to previous study, we identified a significant proportion of MDR *E.coli* following urological procedures, thus underscoring the importance of implementing appropriate antibiotic prophylaxis.^1–4,7,9,11,12^ In our study, the rate of postoperative infection was varied by type of procedures and ranged from 7.2% (TURP) to 30.9% (PCNL). In Thailand, the previous studies have been reported rate of postoperative infection ranged from 7.8% (URS) to 19.4% (transurethral anatomical enucleation of prostate).^11,12^

There were some limitations in this study. First, this study was performed in a single hospital, which may limit generalizability of preoperative antibiotic prophylaxis. Second, we used a retrospective study design. The history of recent hospital admissions, antibiotic use and prior UTIs data outside the TUH might be unavailable. Third, the prevalence of MDR pathogens and antibiotic prophylaxis regimen would be varies in other areas based on the regional or hospital specific antibiogram data. Finally, the small sample size in this study limited our power to detect changes in outcome results.

In conclusion, CKD and the RCRI score were novel risk factors and associated with postoperative infections following urological procedures. The increasing prevalence of MDR pathogens highlighted the need for new strategies or appropriate antibiotic prophylaxis. A future study to identified additional risk factors for MDR bacterial infections should be conducted and prioritized rectal screening before urological procedures in patients with negative urine culture screening.

## Supporting information

10.1017/ash.2025.10271.sm001 Tantiwattanapaibul et al. supplementary material Tantiwattanapaibul et al. supplementary material

## Supplementary material

The supplementary material for this article can be found at https://doi.org/10.1017/ash.2025.10271.

## References

[1] Zhang H , Jiang T , Gao R et al. Risk factors of infectious complications after retrograde intrarenal surgery: a retrospective clinical analysis. J Int Med Res 2020;48:300060520956833. 32993406 10.1177/0300060520956833 PMC7536499

[2] Lin J , Yang Z , Ye L et al. Pathogen species are the risk factors for postoperative infection of patients with transurethral resection of the prostate: a retrospective study. Sci Rep 2023; 13:20943. 38016988 10.1038/s41598-023-47773-7 PMC10684857

[3] Yang Z , Lin D , Hong Y et al. The effect of preoperative urine culture and bacterial species on infection after percutaneous nephrolithotomy for patients with upper urinary tract stones. Sci Rep 2022; 12:4833. 35318408 10.1038/s41598-022-08913-7 PMC8941140

[4] Dybowski B , Bres-Niewada E , Rzeszutko M et al. Risk factors for infectious complications after retrograde intrarenal surgery – a systematic review and narrative synthesis. Cent Eur J Urol 2021;74:437– 445. 10.5173/ceju.2021.250 PMC8552946 34729234

[5] Lai WS , Assimos D. Factors associated with postoperative infection after percutaneous nephrolithotomy. Rev Urol 2018;20:7– 11. 29942195 10.3909/riu0778 PMC6003297

[6] Elsaqa M , Dowd K , El Mekresh A , Doersch KM , El Tayeb MM. Predictors of postoperative urinary tract infection following holmium laser enucleation of the prostate. Can Urol Assoc J 2023;17:E364– E368. 37549346 10.5489/cuaj.8269 PMC10657227

[7] Dumford D 3rd, Suwantarat N , Bhasker V. Outbreak of fluoroquinolone-resistant *Escherichia coli* infections after transrectal ultrasound-guided biopsy of the prostate. Infect Control Hosp Epidemiol 2013;34:269– 73. 23388361 10.1086/669512

[8] Zaytoun OM , Vargo EH , Rajan R , Berglund R , Gordon S , Jones JS. Emergence of fluoroquinolone-resistant *Escherichia coli* as cause of postprostate biopsy infection: implications for prophylaxis and treatment. Urology 2011;77:1035–1041. 21420152 10.1016/j.urology.2010.12.067

[9] Son Y , Dalton R , Daidone C et al. Preoperative and intraoperative urine cultures and its association with postoperative infection after ureteroneocystostomy. Urology 2024;183:176– 184. 37774848 10.1016/j.urology.2023.09.003

[10] Reich DA , Adiyeke E , Ozrazgat-Baslanti T et al. Clinical considerations for patients experiencing acute kidney injury following percutaneous nephrolithotomy. Biomedicines 2023 11:1712. 37371807 10.3390/biomedicines11061712 PMC10296554

[11] Kittaweerat N , Buaban K , Tansakul P. Postoperative infection after ureterorenoscopic lithotripsy in Songkhla Hospital. Insight Urol 2023;44:68– 74.

[12] Sa-nguancharoenpong J , ThaidumrongInsight T. Association between the levels of postoperative pyuria and urinary tract infection in patients undergoing Transurethral Anatomical Enucleation of Prostate (TUAEP) in Rajavithi hospital.Insight Urol 2023;44:1– 6.

