# Supplementary material for: Risk factors for infections after urological procedures among patients with negative urine culture screening
Source: Antimicrob Steward Healthc Epidemiol. 2026 Jan 20;6(1):e26. doi: 10.1017/ash.2025.10271 (PMC12835951; doi:10.1017/ash.2025.10271)
Supplement: Tantiwattanapaibul et al. supplementary material [file S2732494X25102714sup001.docx]

**Supplement**

**Supplement: Multivariate regression analysis risk factor of extended spectrum cephalosporinase pathogens detected in urine culture in infected group**

| Variable | Multivariate analysis | |
| --- | --- | --- |
|  | OR | *P*-value |
| Chronic kidney disease stage III-V | 3.06 (0.52-18.01) | 0.22 |
| History of prior UTI in past 3 months | 0.71 (0.15-3.45) | 0.67 |
| Metabolic equivalents score ≥ 4 | 0.35 (0.03-4.67) | 0.43 |
| Percutaneous nephrolithotomy | 0.86 (0.16-4.61) | 0.86 |
| Transrectal ultrasound and biopsy of prostate | 4.22 (0.33-54.28) | 0.27 |
| Holmium laser enucleation of the prostate | 2.92 (0.36-23.94) | 0.32 |
|  |  |  |

Abbreviations: UTI urinary tract infection
